# Assessment of adaptive immune responses of dairy cows with *Burkholderia contaminans*-induced mastitis

**DOI:** 10.3389/fmicb.2023.1099623

**Published:** 2023-03-07

**Authors:** Zhipeng Zhang, Yiyang Yao, Jiayu Yang, Hui Jiang, Ye Meng, Wenqiang Cao, Fuzhen Zhou, Kun Wang, Zhangping Yang, Chunhua Yang, Jie Sun, Yi Yang

**Affiliations:** ^1^Jiangsu Co-innovation Center for Prevention and Control of Important Animal Infectious Diseases and Zoonoses, College of Veterinary Medicine, Yangzhou University, Yangzhou, China; ^2^College of Animal Science and Technology, Yangzhou University, Yangzhou, China; ^3^Joint International Research Laboratory of Agriculture and Agri-Product Safety, Ministry of Education, Yangzhou University, Yangzhou, China; ^4^Institute of Biological Resources, Jiangxi Academy of Sciences, Nanchang, China; ^5^Shenzhen Academy of Inspection and Quarantine Sciences, Shenzhen, China

**Keywords:** dairy cows, adaptive immune responses, *Burkholderia contaminans*, mastitis, flow cytometry

## Abstract

*Burkholderia contaminans*, an emerging pathogen related to cystic fibrosis, is known to cause potentially fatal infections in humans and ruminants, especially in immunocompromised individuals. However, the immune responses in cows following its infection have not been fully elucidated. In this study, T- and B-lymphocytes-mediated immune responses were evaluated in 15 *B. contaminans*-induced mastitis cows and 15 healthy cows with multi-parameter flow cytometry. The results showed that infection with *B. contaminans* was associated with a significant decrease in the number and percentage of B lymphocytes but with a significant increase in the proportion of IgG^+^CD27^+^ B lymphocytes. This indicated that humoral immune response may not be adequate to fight intracellular infection, which could contribute to the persistent bacterial infection. In addition, *B. contaminans* infection induced significant increase of γδ T cells and double positive (DP) CD4^+^CD8^+^ T cells but not CD4^+^ or CD8^+^ (single positive) T cells in blood. Phenotypic analysis showed that the percentages of activated WC1^+^ γδ T cells in peripheral blood were increased in the *B. contaminans* infected cows. Interestingly, intracellular cytokine staining showed that cattle naturally infected with *B. contaminans* exhibited multifunctional TNF-α^+^IFN-γ^+^IL-2^+^*B. contaminans*-specific DP T cells. Our results, for the first time, revealed a potential role of IgG^+^CD27^+^ B cells, CD4^+^CD8^+^ T cells and WC1^+^ γδ T cells in the defense of *B. contaminans*-induced mastitis in cows.

## Introduction

*Burkholderia* are non-spore-forming, obligately aerobic, rod-shaped, Gram-negative bacteria that are ubiquitously found in plants, animals, soil, and water (Rhodes and Schweizer, 2016). This genus contains some common pathogens, such as *Burkholderia cepacia*, *Burkholderia pseudomallei*, and *Burkholderia mallei* (Foxfire et al., 2021). It is recognized that these bacteria may cause potentially deadly infections in humans and/or ruminants, particularly in immunocompromised individuals (Limmathurotsakul et al., 2014). Generally, transmission of the disease occurs as a result of the exposure to the water or soil where the organisms ordinarily live (Zheng et al., 2021). In 2009, *Burkholderia contaminans*, an emerging pathogen linked to cystic fibrosis, was included in the *B. cepacia* complex group (Vanlaere et al., 2009). *Burkholderia contaminans* has remarkable ability to synthesize antifungal chemicals and survive in a polymicrobial environment (Bernier et al., 2016). After being struck by a cow’s tail in the right eye, a patient developed redness and discomfort for over 20 days, and *B. contaminans* was isolated from the secretions of that eye. Additional clinical examination revealed the presence of fungal ulcer in the attacked eye of the patient (Lama et al., 2021). *Burkholderia contaminans* was also considered to be a novel pathogen of bovine mastitis, implicated in many outbreaks in diverse geographic regions (Wang et al., 2022), although it has been usually disregarded. Multidrug-resistant *B. contaminans* has been identified with multiple sources of antimicrobial resistance genes. The big G + C-rich genome contains a multitude of virulence factors, emphasizing its pathogenicity (Alnoch et al., 2019). This presents new challenges for the prevention and treatment of bovine mastitis.

Bovine mastitis has become the most widespread and expensive production disease in dairy herds worldwide, which is usually caused by intramammary bacterial infection (Seegers et al., 2003). While better dairy herd procedures have helped to eradicate many Gram-positive pathogens that induce mastitis, they have been mostly unsuccessful in reducing the frequency of intramammary infections induced by Gram-negative bacteria (Bannerman et al., 2005). *Escherichia coli* is the most frequent Gram-negative bacteria that cause mastitis in cattle, and most of our knowledge of the innate immune response to Gram-negative infection comes from previous studies in this bacterium (Zaatout, 2022). In contrast, the adaptive immune response to other widespread Gram-negative bacteria, such as *B. contaminans*, is far less understood.

Bovine adaptive immune responses consist of cellular and antibody-mediated immune responses that are primarily driven by lymphocytes (Vlasova and Saif, 2021). Different γδ and αβ T-cell subsets are implicated in the protection of the breast against mastitis, and the activation of these T-cell subsets varies among pathogens (Soltys and Quinn, 1999). It was previously found that the proportion and expression of B-cells increased in blood of dairy cows with chronic sub-clinical mastitis after *Staphylococcus aureus* infection (Grönlund et al., 2006). Since certain pathogens may infiltrate and survive intracellularly, a selective activation of B-cells, indicating the establishment of humoral response, might not be adequate to eradicate intracellular bacteria, which may explain the persistence of infection. Nevertheless, the contributions of diverse lymphoid populations to host defense in spontaneously infected mammary glands of cows remains to be thoroughly investigated. During a bacterial infection, both leukocyte adhesion and the production of cytokines play important roles (Li et al., 2018). However, the proportional contributions of these variables to the pathogenesis of mastitis are unclear and more research is needed.

While most previous studies have been documented the innate immune response to *E. coli*, less is understood regarding the acquired immunological response of the mammary gland to other Gram-negative bacteria, such as *B. contaminans*, which has been demonstrated to causes mastitis in cows. We detected an outbreak of cow mastitis caused by single *B. contaminans* at a dairy farm in Jiangsu province, China, with a total of 20 cases. The purpose of this study was to undertake assessment of T and B cell immune responses against *B. contaminans* naturally-induced mastitis in dairy cows to develop better strategy against very virulent *B. contaminans* strains. In the research presented here, we examined the idea that various B and T lymphocyte subgroups are collectively implicated in the udder’s response to a mastitis infection and release pathogen-specific cytokines or antibodies. To test this theory, we have employed a panel of monoclonal antibodies to define the lymphocyte subsets in mastitis and normal peripheral blood in terms of lymphocyte subsets distribution, cytokines, adhesion molecule, and antibody expression. Furthermore, we have presented a comparison of these characteristics for naturally infected cows with *B. contaminans*, as well as a correlation study between parameters and colony-forming units. These investigations have helped to define the function of the distinct lymphocyte subgroups in the host defense of the cow mammary gland toward infection with *B. contaminans*, a Gram-negative bacterium that causes mastitis but is clinically significant.

## Materials and methods

### Animals

All experimental methods in this work were evaluated and approved by Yangzhou University Institutional Animal Care and Use Committee (JBGS2022-SYXY-2). This research was conducted in conformity with the Administration of Affairs Concerning Experimental Animals issued by the Chinese Ministry of Science and Technology.

This study was conducted in a well-governed dairy farm in Jiangsu province, China. There were a total of 1,535 cows in this farm, of which 650 were in lactation. In this farm, lactating cows were kept in free stalls and utilized sawdust as bedding. The stalls were cleaned twice per week. Once a day, the feces were eliminated from the sawdust, and enough new sawdust was introduced to replace the manure-containing sawdust. The cows were milked in rotating milking parlors following the same suggested milking procedure, which included examining the udder and foremilk, disinfecting the teats by dipping.

Prior to this study, 20 lactating cows were diagnosed with clinical mastitis by the farm veterinarians. A case of clinical mastitis was characterized by clinical symptoms, such as abnormal udder (red, swollen, or hard), abnormal milk, or fever (Song et al., 2020). Subsequently, milk samples from these cows with mastitis were collected. The unanimously isolated pathogens was *B. contaminans*. To reduce the influence of factors other than *B. contaminans* natural infection on bovine immune response, we investigated milk yield, pedigree, and dairy herd improvement (DHI) records and carefully selected 15 *B. contaminans* naturally-induced mastitis cows as the test group. Fifteen healthy cows in the control group were selected according to the same criteria of test group cows. Before the diagnosis of mastitis, these Holstein dairy cattle all had the same lactation number and had comparable individual performance and ages (Table 1). In addition, with commercial ELISAs (Qiaodu, Shanghai, China) or PCRs (Yang et al., 2022), it was preliminarily determined that none of the experimental cows infected any of the following pathogens: *S. aureus* (Yang and Laven, 2022), *Bacillus cereus* (Simbotwe et al., 2018), *Streptococcus agalactiae* (Bu et al., 2015), *Babesia* spp., *Brucella abortus*, *Anaplasma* spp., or *Theileria* spp.

**Table 1:** Comparison of age, average daily milk yield, SCS, milk composition of cows in the healthy and mastitis groups before the *Burkholderia contaminans* infection.

Cow number	Age, month	Milk yield, kg/day	SCS	Fat, %	Protein, %	Lactose, %
Health group						
16207	42	18.21 ± 3.12	2.12 ± 1.01	3.71 ± 0.43	3.01 ± 0.35	5.44 ± 0.21
17309	38	21.26 ± 2.15	2.23 ± 2.11	3.74 ± 0.13	3.60 ± 0.35	5.65 ± 0.21
17312	38	20.35 ± 2.33	2.31 ± 1.98	3.34 ± 0.22	3.43 ± 0.31	5.67 ± 0.26
17322	35	20.44 ± 4.21	2.11 ± 1.77	3.28 ± 0.31	3.47 ± 0.21	5.50 ± 0.23
17326	36	23.12 ± 3.15	2.01 ± 1.99	3.44 ± 0.35	3.82 ± 0.40	5.36 ± 0.24
17336	37	24.56 ± 4.63	2.06 ± 1.45	3.32 ± 0.19	3.53 ± 0.39	5.21 ± 0.28
17348	37	24.89 ± 4.68	2.17 ± 2.10	3.57 ± 0.40	3.64 ± 0.38	5.46 ± 0.27
17365	37	19.76 ± 5.13	2.13 ± 1.99	3.12 ± 0.21	3.50 ± 0.30	5.42 ± 0.26
17370	37	22.10 ± 3.11	2.88 ± 1.89	3.74 ± 0.44	3.51 ± 0.26	5.48 ± 0.28
17394	37	23.68 ± 4.42	2.00 ± 1.79	3.24 ± 0.31	3.51 ± 0.31	5.60 ± 0.26
17411	38	21.53 ± 4.10	2.41 ± 1.01	3.65 ± 0.41	3.74 ± 0.24	5.56 ± 0.19
17417	38	25.13 ± 4.55	2.46 ± 2.10	3.62 ± 0.42	3.46 ± 0.29	5.04 ± 0.18
17421	38	25.89 ± 5.10	2.89 ± 1.78	3.68 ± 0.41	3.70 ± 0.29	5.08 ± 0.11
18091	31	21.32 ± 2.13	2.77 ± 1.88	3.49 ± 0.31	3.52 ± 0.21	5.42 ± 0.23
18118	31	24.66 ± 2.86	2.13 ± 1.67	3.32 ± 0.21	3.32 ± 0.35	5.62 ± 0.18
Mastitis group						
16105	45	18.44 ± 5.21	1.89 ± 1.03	3.41 ± 0.23	3.65 ± 0.22	5.64 ± 0.22
16203	44	18.64 ± 4.66	2.46 ± 1.02	3.34 ± 0.19	3.14 ± 0.26	5.39 ± 0.23
16229	44	19.14 ± 3.21	2.34 ± 1.33	3.67 ± 0.36	3.16 ± 0.21	5.49 ± 0.23
17022	38	20.78 ± 4.56	2.13 ± 1.43	3.72 ± 0.41	3.56 ± 0.29	5.47 ± 0.29
17032	38	22.89 ± 5.03	1.97 ± 1.56	3.71 ± 0.48	3.35 ± 0.20	5.25 ± 0.21
17146	38	21.46 ± 4.44	2.10 ± 2.00	3.29 ± 0.11	3.17 ± 0.21	5.35 ± 0.11
17181	38	27.13 ± 5.08	2.13 ± 2.07	3.77 ± 0.46	3.46 ± 0.21	5.65 ± 0.30
17194	37	26.15 ± 5.88	2.16 ± 2.05	3.77 ± 0.44	3.51 ± 0.30	5.43 ± 0.19
17220	37	23.11 ± 2.11	2.15 ± 1.89	3.40 ± 0.27	3.47 ± 0.29	5.02 ± 0.11
17369	37	24.56 ± 2.99	2.22 ± 1.99	3.25 ± 0.21	3.15 ± 0.16	5.33 ± 0.13
17383	37	24.11 ± 4.32	2.31 ± 1.78	3.46 ± 0.18	3.18 ± 0.18	5.09 ± 0.10
17404	37	20.46 ± 4.68	2.34 ± 1.73	3.04 ± 0.16	3.28 ± 0.16	5.25 ± 0.21
18083	31	21.45 ± 3.88	2.65 ± 1.52	3.65 ± 0.32	3.04 ± 0.11	5.60 ± 0.26
18205	31	22.45 ± 3.78	2.34 ± 1.44	3.47 ± 0.38	3.12 ± 0.22	5.10 ± 0.14
18271	31	24.65 ± 4.66	2.29 ± 1.33	3.74 ± 0.39	3.67 ± 0.41	5.27 ± 0.22
Variance analysis between the health and mastitis groups						
Value of *p*	0.09	0.59	0.09	0.72	0.72	0.95

The Health and Mastitis groups were compared using unpaired t-test and error bars show the mean ± SD. There were not any significant differences (*p* < 0.05). The data in the table included comprehensive data of each cow for 3 months (March, July, and September 2021), and the data were collected up to 5 months before the point at which each cow was diagnosed with mastitis.

### Microbiological analysis

Mastitis milk samples collection was performed as described previously (Duse et al., 2021). Briefly, teats were submerged in a pre-milking teat disinfection (Kangmu, Hefei, China) for 30 s before being scrubbed with 70 percent alcohol and left to dry. Then, several foremilk streams were eliminated before the sample collection. Simultaneously, a sample of sterile milk was obtained from one quarter of each cow. Within 1 h, milk samples were transported in ice packs to the laboratory in Yangzhou University for bacterial culture. 100 μL of milk from each sample was put onto a culture plate of Columbia Blood agar (Hopebio, Qingdao, China) containing 5 percent defibrillated sheep blood (Jiulong, Zhengzhou, China) upon arrival in the laboratory. Next, these culture plates were incubated for 24 h at 37°C. Following 24 h, the number of colonies on each of the culture plate was determined. All of the colonies were subjected to Gram staining and microscopy. In order to identify potential environmental origins of *B. contaminans*, the samples were collected from many locations in the farm. The 18 samples included milking machine swabs, bovine feed, non-chlorinated and chlorinated water sources, and bovine feces. Microbiology operations are carried out in accordance with published guidelines (Bi et al., 2016).

In order to facilitate bacterial identification, the sample was diluted in a 10-fold gradient until there were fewer than 10 colonies on each culture plate. Based on the morphology of colonies, one more optional sub-culture was conducted if different morphological colonies were observed on a single plate. After the extraction of DNA, bacterial *16S rDNA* genes were amplified utilizing universal primers 27 F (5′-AGAGTTTGATCCTGGCTCAG-3′) and 1492R (5′-GGTTACCTTGTTACGACTT-3′; Alnakip et al., 2020). PCR products were delivered to a commercial lab (Qingke, Shanghai, China) for *16S rDNA* sequencing, and the species of bacteria were determined by BLASTN with the assembled sequences. In addition, MEGA software (Mega Limited, MEGA 7) was used to perform evolutionary analysis for the sequences identified as *B. contaminans* (Kumar et al., 2018). The detected colonies were then regularly cultivated at 37°C in brain-heart infusion broth (Cloolaber, Beijing, China) and kept at −80°C in 30% of glycerol.

### The isolation of mononuclear leucocytes in blood

As described previously, clinical symptoms of bacteria-induced mastitis generally appear on the 7th day after infection, and the lymphocyte immune response is most evident on the 14th day (Bannerman et al., 2005). Therefore, whole blood samples were collected from jugular vein at the 7th day after the diagnosis of mastitis. Peripheral blood mononuclear cells (PBMCs) were purified from anticoagulated blood by density gradient centrifugation (650 × g, 35 min) using lymphocyte separation medium (TBD science, Tianjin, China), and then re-suspended in the complete medium (RPMI-1640 with 1% antibiotics and 10% FBS) as previously reported (Cabanelas et al., 2020). The isolated PBMCs were utilized immediately for multiparameter flow cytometry analysis and cryopreserved (−80°C) for future *in vitro* investigations.

### Phenotypic analysis of PBMCs

Freshly extracted PBMCs were suspended in FACS buffer (0.5% FBS in PBS) and calibrated to 2 × 10^6^ cells per sample for phenotypic analysis of T and B cells. Table 2 lists the secondary reagents and monoclonal antibodies (mAbs) utilized for cell surface staining. Cells were stained in V-bottom plates and incubated for 20 min at 37°C with 1% bovine serum to inhibit FC receptors (Hao et al., 2021). Then, the cells were washed with FACS buffer by centrifuging at 1,600 rpm for 5 min at 4°C. At least 1 × 10^5^ cells were collected for flow cytometry analysis.

**Table 2:** Antibodies used for flow cytometry in this study.

Marker	Clone	Isotype	Conjugate	Labeling strategy	Labeling strategy
B cells					
CD21	CC21	IgG1	PE	Direct conjugate	Bio-rad
IgG	AB_429708	IgG	FITC	Direct conjugate	Invitrogen
CD27	M-T271	IgG1, κ	BrilliantViolet510	Direct conjugate	BioLegend
T cells					
CD3	MM1A	IgG_1_	PerCP-Cy5.5	Secondary antibody	Kingfisher
WC1	CC15	IgG2a	FITC	Direct conjugate	Bio-rad
CD4	CC8	IgG2a	AlexaFluor647	Direct conjugate	Bio-rad
CD8	CC63	IgG2a	PE	Direct conjugate	Bio-rad
CD27	M-T271	IgG1, κ	BrilliantViolet510	Direct conjugate	BioLegend
CD44	IMC	IgG2b, κ	PrestoBlue	Direct conjugate	BioLegend
Triple cytokine staining					
CD3	MM1A	IgG_1_	PerCP-Cy5.5	Secondary antibody	Kingfisher
CD4	CC8	IgG2a	AlexaFluor647	Direct conjugate	Bio-rad
CD8	CC63	IgG2a	PE-Cy7	Secondary antibody	Bio-rad
TNF-α	CC327	IgG2b	AlexaFluor488	Direct conjugate	Bio-rad
IFN-γ	CC302	IgG_1_	PE	Direct conjugate	Bio-rad
IL-2	MT3B3	IgG_1_	BV421	Biotin-streptavidin	Mabtech

### Intracellular cytokine staining

In order to intracellularly stain CD8^+^ and CD4^+^ T lymphocytes with antibodies of TNF-α, IFN-γ, and IL-2, PBMCs were calibrated to 3 × 10^6^ cells per well in 150 μL complete medium. Subsequently, cells were stimulated with *B. contaminans* at 37°C for 18 h (infection strain, MOI = 0.1). Brefeldin A (BD Biosciences, San Jose, United States) was maintained in micro-cultures at a final 1 μg/mL concentration throughout the course of 4 h. While the controls were incubated with a mixture medium of ionomycin (500 ng/mL) and phorbol myristate acetate (50 ng/mL). For further flow cytometry (FCM) labeling on microtiter-plates, cells were rinsed in PBS comprising 1% FBS before being incubated with the secondary reagents and antibodies described in Table 2 (Talker et al., 2015). According to the manufacturer’s recommendations, Fixation/Permeabilization Kit (BD Biosciences, Beijing, China) were employed to fix and permeabilize cells.

### Flow cytometry

Flow cytometry was performed with LSRFortessaTM flow cytometer (BD Biosciences, San Jose, United States) outfitted with five lasers (488, 640, 405, 561, and 361 nanometer) and a High Throughput Sampler. Automatic compensation calculations were performed on single-stain samples. At least 1 × 10^5^ lymphocytes were obtained for the study of B and T cell subgroups. 5 × 10^5^–1 × 10^6^ cells were counted in order to identify intracellular cytokines. The tactics for gating were relied on FMO. The data was analyzed using FlowJo software (BD Biosciences, San Jose, United States) and uploaded to Prism 9.4 software (Graphpad, San Diego, United States) for further computations and graph production.

### The quantification of IgG in serum

Serum IgG were quantified using commercial bovine IgG Platinum ELISA kits (Qiaodu, Shanghai, China).

### Statistical analyses

The statistical significance was calculated with *t*-test as independent samples using the SPSS 20.0 software (IBM, Ehningen, Germany). The data is presented as a comparison between the health and mastitis groups. If *p* < 0.05 (*), *p* < 0.01 (**), or *p* < 0.001 (***), the results were considered statistically significant.

## Results

### *Burkholderia contaminans* was identified as pathogenic bacteria in raw milk samples from cows with mastitis

**Figure 1:**
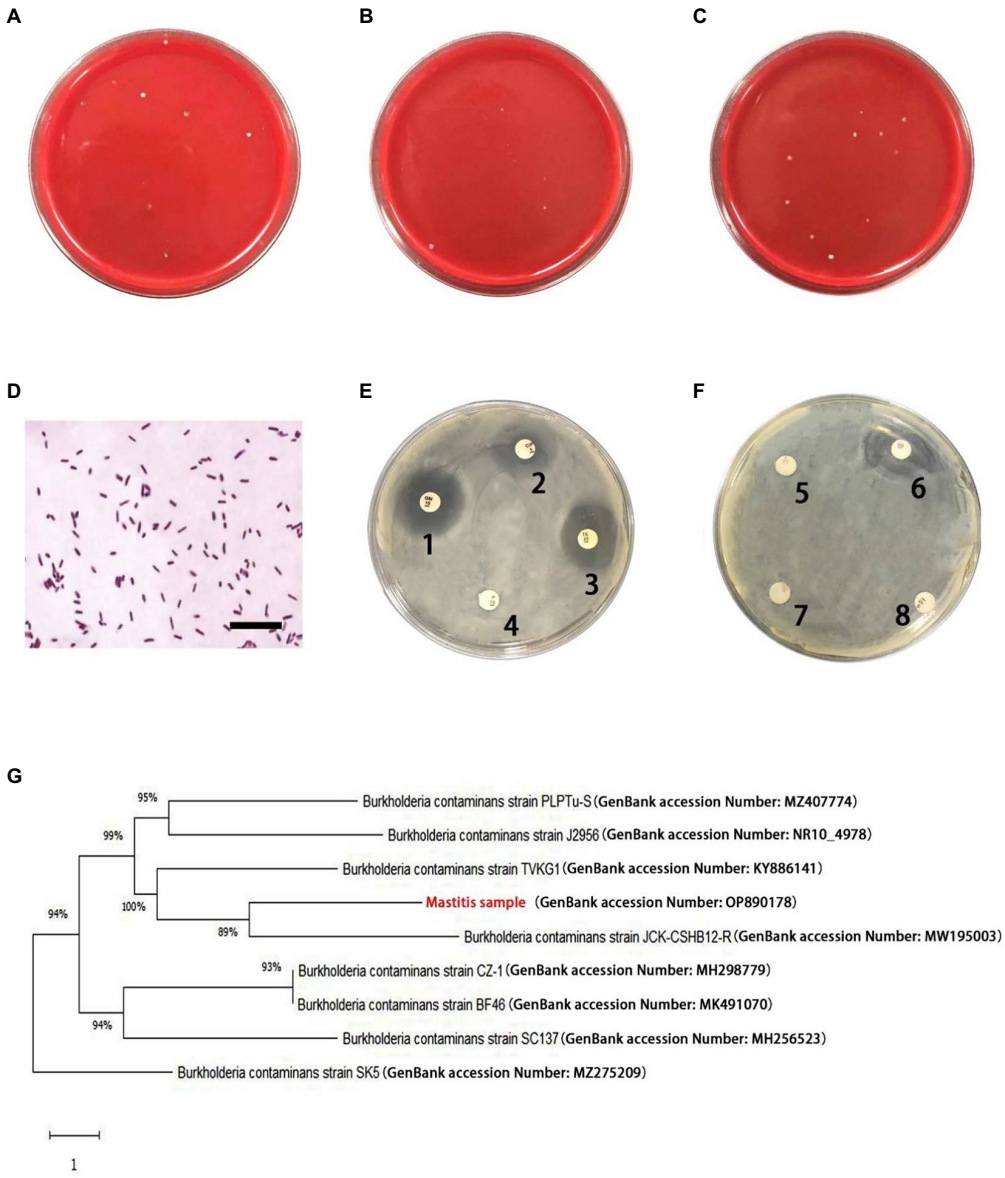
Isolation and identification of pathogenic bacteria. **(A)** Representative plates from undiluted milk samples from healthy cows. **(B)** Representative plates from undiluted environmental samples. **(C)** Representative plates from milk samples (diluted 10 times) with mastitis. **(D)** Gram-positive irregularity rod-shaped bacteria (scale bar is 10 μm). **(E,F)** Antimicrobial resistance profiles of the *Burkholderia contaminans* isolates. The round white papers on the petri dish are antibiotic-sensitive tablets (1: Gentamicin, 2: Ciprofloxacin, 3: Tetracycline, 4: penicillin, 5: lincomycin, 6: Cephalosporin, 7: Streptomycin, 8: Amoxicillin). **(G)** Phylogenetic analysis of *B. contaminans*. Red fonts indicate *B. contaminans* isolates in this study.

There were small amount of bacterial colonies on culture dishes plated with milk from healthy glands and environmental samples (Figures 1A,B). These bacteria were identified as non-mastitis-causing environmental bacteria in normal circumstances, including *Bacillus licheniformis*, *Alcaligenes faecalis*, *Enterobacter cloacae* and *Alfalfa rhizobium.* Among these 20 cows with mastitis, two of them were identified to be co-infected with *B. contaminans* and *Staphylococcus hemolyticus*/ *E. coli*, respectively. While *B. contaminans* was identified as the only pathogenic bacterium in the milk of the remaining 18 cows, although 1–5 strains of environmental bacteria not normally thought to cause mastitis in cows were also isolated, including *Alcaligenes faecalis* and *Alfalfa rhizobium* (Figure 1C). Subsequently, 15 cows were employed in further studies. Table 3 indicated the numbers of colonies in the culture dishes of milk samples from these 15 cows. The bacteria were negative by Gram staining (Figure 1D). *Burkholderia contaminans* was found to be resistant to several tested antibiotics (penicillin, streptomycin, lincomycin and amoxicillin), but not ciprofloxacin, tetracycline, gentamicin and cephalosporin (Figures 1E,F). The *B. contaminans* isolated in this study (red fonts, GenBank accession Number: OP890178) was closely related to the strain isolated from *prunus yedoensis* (*B. contaminans* strain JCK-CSHB12-R, GenBank accession Number: MW195003) in Korea and the strain isolated from human blood in India (*B. contaminans* strain TVKG1, GenBank accession Number: KY886141) (Figure 1G).

**Table 3:** Quantitative detection level for *B. contaminans* in undiluted milk samples.

Cow number	Number of colonies on the medium				Log_10_ CFU/mL
	1st trial	2nd trial	3rd trial	Mean	
16105	126	140	131	132.33	4.12
16203	114	121	101	112.00	4.05
16229	181	194	175	183.33	4.26
17022	199	182	191	190.67	4.28
17032	186	161	175	174.00	4.24
17146	130	118	103	117.00	4.07
17181	76	68	70	71.33	3.85
17194	170	163	179	170.67	4.23
17220	146	144	147	145.67	4.16
17369	176	191	180	182.33	4.26
17383	109	86	91	95.33	3.98
17404	94	120	108	107.33	4.03
18083	91	119	105	105.00	4.02
18205	170	178	186	178.00	4.25
18271	114	101	110	108.33	4.03

### Decreased number and percentage of CD21^+^ B cells in the lymphocytes of cows with mastitis

**Figure 2:**
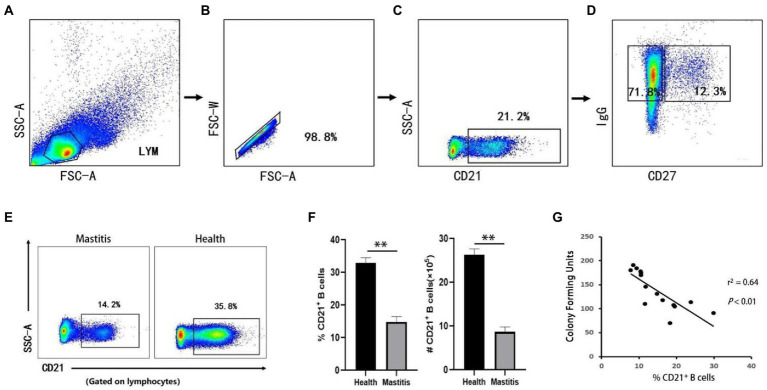
Gating strategies to define bovine B cell subsets and the number and the percentage of B cells in two groups. The lymphocytes are chosen with FSC versus SSC **(A)** and then singlets are gated using FSC-A and FSC-W **(B)**. By outputting CD21, CD21^+^ (B cells) are identified **(C)**. By outputting IgG versus CD27, IgG^+^CD27^−^, IgG^+^CD27^+^ are identified, respectively **(D)**. **(E)** Representative dot plot of CD21^+^ B cells. **(F)** Absolute number and percentage and of CD21^+^ B cells in lymphocytes. **(G)** Relationship of CFU of *B. contaminans* with the percentage of CD21^+^ B cells.

B lymphocytes can serve as antigen-presenting cells, and differentiate into plasma cells that produce immunoglobulins (Wang et al., 2020). In cattle, CD21 is restricted to be expressed in B lymphocytes (Naessens et al., 1990), and can serves as a typical surface marker. A basic gating strategy is shown in Figures 2A–D. The absolute number of CD21^+^ B cells in the peripheral blood samples was determined by FCM based on physical parameters. As shown in Figures 2E,F, the number and the percentage of CD21^+^ B cells in the lymphocyte gate in cows with *B. contaminans*-induced mastitis were significantly lower than those in healthy cows. This suggested that the decreased absolute number of B cells could fully or partially contribute to the decreased percentage of B cells. In addition, the absolute number of CD21^+^ B cells in the peripheral blood was determined to be negatively associated with the CFU of the milk samples (*p* = 0.002, *r*^2^ = 0.641; Figure 2G).

### Increased percentage of IgG^+^CD27^+^ B cells in the cows with mastitis

**Figure 3:**
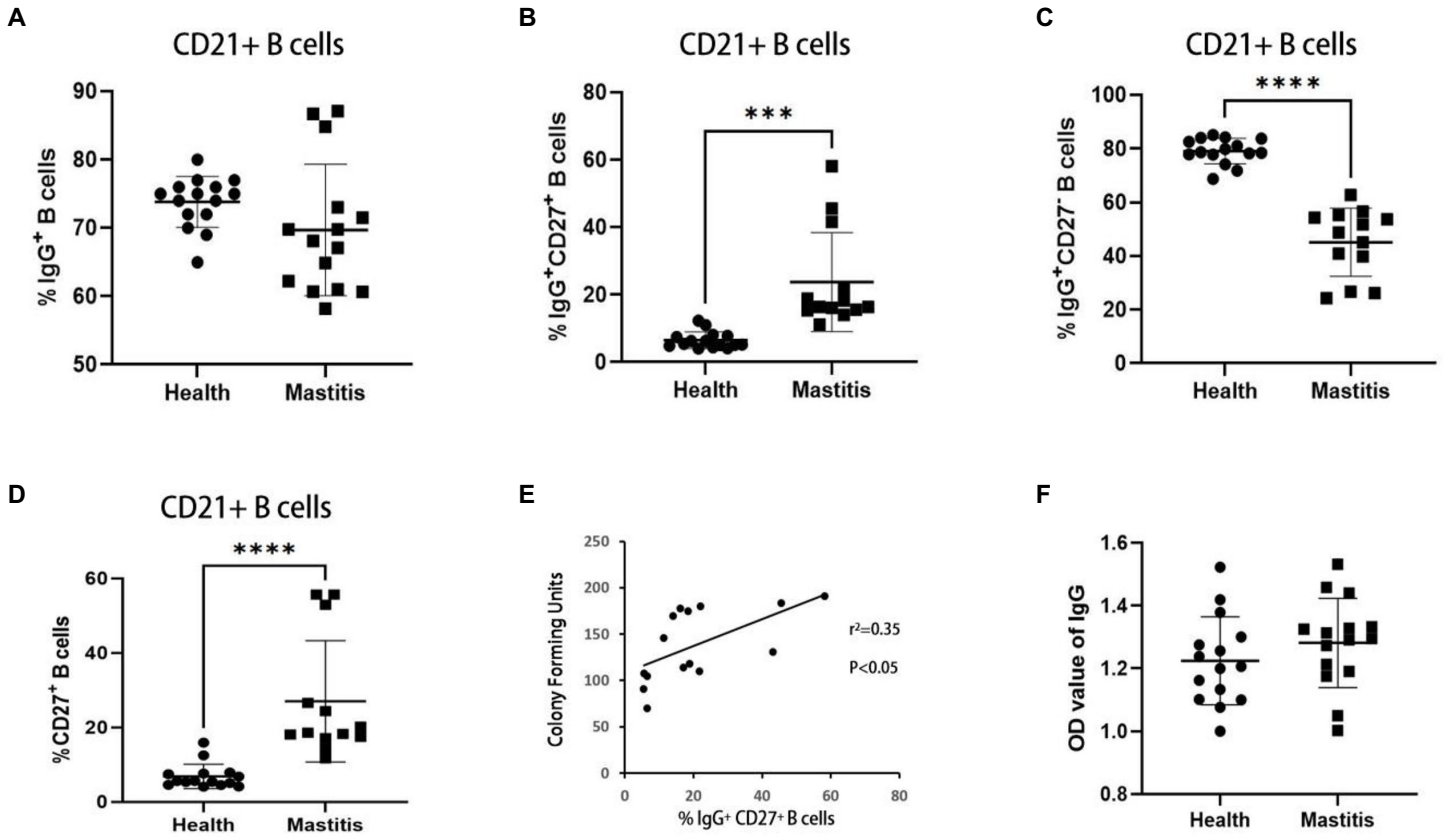
The cows of IgG^+^ CD27^+^ B cells increased in the mastitis patients. Percentages of IgG^+^ **(A)**, IgG^+^CD27^−^ **(B)** and IgG^+^CD27^+^ B cells **(C)** in CD21^+^ B cells. **(D)** Percentage of memory B cells (CD27^+^ B cells) in CD21^+^ B cells. **(E)** Relationship of CFU of *B. contaminans* with the percentage of IgG^+^CD27^+^ B cells. **(F)** Expression of serum total IgG antibody in the two groups.

IgG^+^ B cells can develop into IgG antibody-secreting cells, which are associated with antigen-specific IgG memory B cell responses (Chen et al., 2015). According to studies conducted in cattle, infection with *M. bovis* leads to the formation of IgG memory B cells, which may quickly convert into antibody-producing plasma cells upon re-exposure to the antigen (Lyashchenko et al., 2020). IgG^+^ B cells were subdivided into IgG^+^CD27^−^ B cells and IgG^+^CD27^+^ B cells based on CD27 expression (Kong et al., 2016), a handy marker of memory B cells. In this study, the effects of *B. contaminans* infection on IgG^+^ B cells (IgG^+^ CD21^+^ lymphocytes) were characterized using PBMCs from 15 *B. contaminans*-infected cows and 15 healthy cows. While the percentage of IgG^+^ B cells in the *B. contaminans*-infected cows was not significantly different from that in the healthy cows (Figure 3A), the proportion of IgG^+^CD27^+^ B cells were significantly increased in *B. contaminans*-infected cows (Figures 3A–C). Since IgG^+^CD27^+^ B cells are part of the memory B cell compartment, we evaluated the percentage of memory B cells in both groups, and the results indicated that the proportion of memory (CD27^+^) B cells was higher in the *B. contaminans*-infected cows (Figure 3D). Analysis of correlation revealed that the proportion of IgG^+^CD27^+^ B cells in *B. contaminans*-infected cows was positively associated with the CFU of *B. contaminans* (Figure 3E). There was no statistically significant difference in the levels of IgG antibodies in *B. contaminans*-infected and healthy cows (Figure 3F).

### *Burkholderia contaminans* infection induced significant increase of WC1^+^ γδ T cells and CD4^+^CD8^+^ DP T cells in cows with mastitis

**Figure 4:**
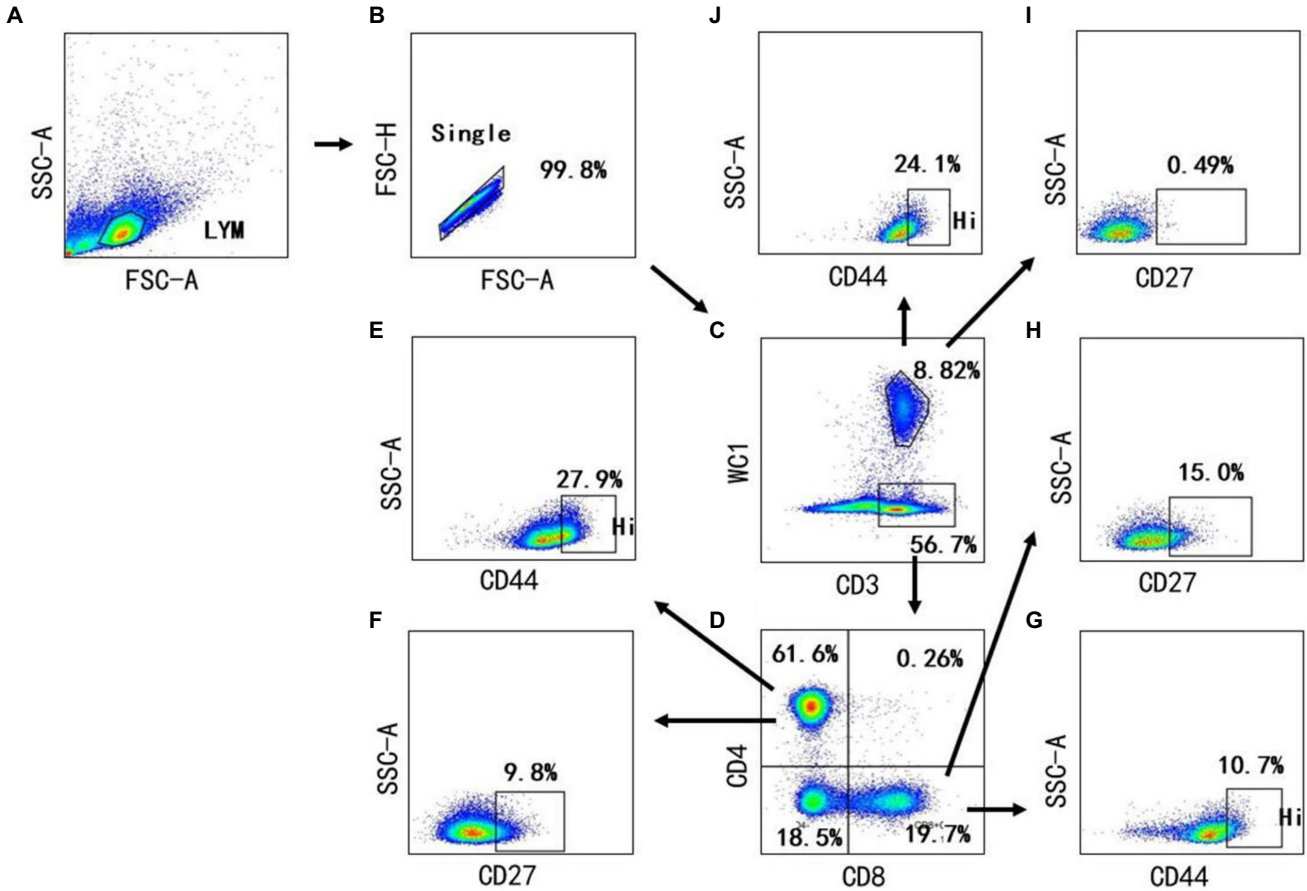
Gating strategies to define bovine T cell subsets. Mononuclear cells were isolated from blood of cows and surface stained with antibody cocktails (CD3, CD4, CD8, WC1, CD44, CD27). The lymphocytes are chosen with FSC versus SSC **(A)** and then singlets are gated using FSC-A and FSC-H **(B)**. By outputting CD3 versus WC1, WC1^−^ CD3^+^ T cells, WC1^+^ γδ T cells are identified, respectively **(C)**. By outputting CD4 versus CD8 in the WC1^−^ CD3^+^ T cells, CD4^+^ CD8^−^, CD8^+^ CD4^−^ and CD8^+^ CD4^+^are identified, respectively **(D)**. The CD4^+^ CD8^−^ T cells contains CD44^+^ **(E)** and CD27^+^ T cells **(F)**. CD8^+^ CD4^−^ T cells contain CD44^+^ **(G)** and CD27^+^ T cells **(H)**. The WC1^+^ γδ T cells contains CD27^+^ **(I)** and CD44^+^ γδ T cells **(J)**.

**Figure 5:**
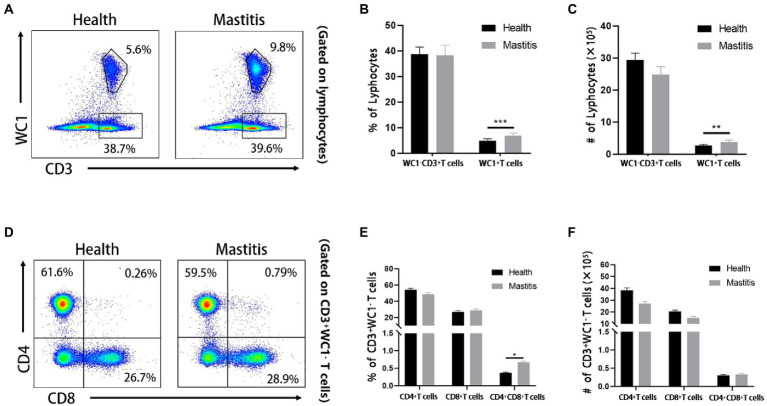
Phenotypic analysis of T cell subsets of cows of different groups. **(A)** Representative dot plots of WC1^+^ γδ T cells and WC1^−^ CD3^+^ T cells from two groups. Percentage **(B)** and absolute number **(C)** of WC1^−^ CD3^+^ T and WC1^+^ γδ T cells in lymphocytes. **(D)** Representative dot plots of bovine CD4^+^ CD8^−^, CD8^+^ CD4^−^ and CD8^+^ CD4^+^ T cells from healthy and mastitis groups. Percentage **(E)** and absolute number **(F)** of CD4^+^, CD8^+^ and CD8^+^ CD4^+^ DP T cells in WC1^−^ CD3^+^ T cells.

Cellular immunologic response is deemed to play a crucial role in the prevention of *Burkholderia* (Barnes et al., 2004). Nevertheless, the changes of diversified T cell subsets in the bovine peripheral blood have not been well-characterized after infection with *Burkholderia*. Therefore, we used FCM to analyze the changes of CD4, CD8, and γδ T cells of cows with *B. contaminans* naturally-induced mastitis. The gating strategy was shown in Figure 4. As depicted in typical dot-plots (Figure 5A), compared with the healthy cows, *B. contaminans* infection induced significantly higher absolute number and percentage of WC1^+^ γδ T cells in the blood. On the contrary, there was no statistical difference in the absolute number and percentage of WC1^−^CD3^+^ T cells between the health and mastitis groups (Figures 5B,C).

Although there was no significant difference in WC1^−^CD3^+^ T cells of cows infected with *B. contaminans*, compared to the healthy cows, it was not clear whether the changes in diversiform T cell subsets were different between the two groups. We distinguished four T cell subsets (CD4^+^, CD8^+^, CD4^−^CD8^−^ and CD4^+^CD8^+^) among WC1^−^CD3^+^ T cells (Figure 5D) and found that *B. contaminans* infection of mammary gland elicited significantly higher percentage of CD4^+^CD8^+^ DP T cells in the blood, compared to the health group (Figures 5E,F). There was no difference in the absolute number and percentage of CD4^+^ and CD8^+^ T cells in the blood between the two groups.

### Increased activation, but not differentiation, of γδ T cells in cows with mastitis

**Figure 6:**
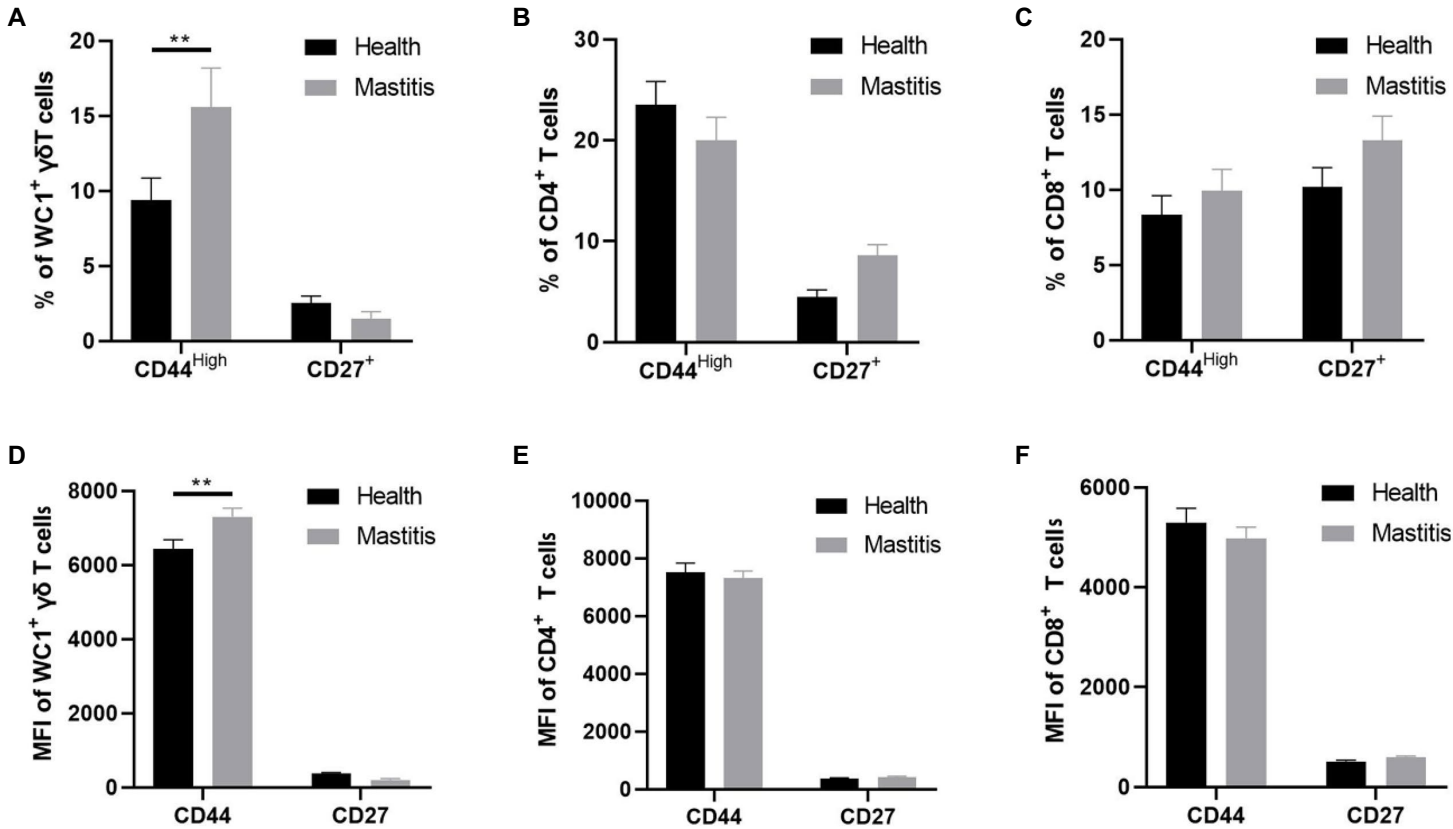
Difference of CD44 and CD27 expression in different subsets of T cells (CD4^+^, CD8^+^, WC1^+^) of health and mastitis calves. Representative histograms show CD44^high^ and CD27 expression on CD4^+^CD8^−^ **(A)**, CD4^−^ CD8+ **(B)** T cells and WC1^+^γδ T cells **(C)** from the blood of health and mastitis calves. Bar graphs show MFI of CD44 and CD27 on CD4^+^ **(D)**, CD8^+^ T cells **(E)** and WC1^+^γδ T cells **(F)** from the two groups.

The activation and differentiation of T cells are often impaired in chronic infection (Dimeloe et al., 2017). CD27 is a co-stimulatory molecule that is commonly used to identify the differentiation stage of T cells (Guerra-Maupome et al., 2019). We detected the percentages and mean fluorescence intensity (MFI) of CD27^+^ T cells in the T subsets of cows with mastitis and healthy cows, but found no differences in the percentages and MFI of CD27^+^ T cells in either WC1^+^ γδ, CD8^+^ or CD4^+^ T cells between the two groups (Figure 6). These results indicated that T cells from the *B. contaminans* infected cows shared a similar differentiation phenotype with those from the healthy cows at early stage of infection.

Like integrin expression, CD44 expression is upregulated after lymphocyte activation, thereby facilitating its movement through the extracellular matrix through interactions with hyaluronic acid and fibrin (Bosworth et al., 1991). To investigate the activation of T subsets, we detected the expression of CD44 molecule in the T subsets of cows with mastitis and healthy cows. The results showed that the percentages of activated WC1^+^ γδ T cells in peripheral blood were increased in the *B. contaminans* infected cows. However, there was no significant difference in the percentage of CD4^+^CD44^High^ and CD8^+^CD44^High^ T cells in the blood between the two groups (Figure 6).

### *Burkholderia contaminans* induced synchronous secretion of triple cytokine (TNF-α, IFN-γ, and IL-2) in CD4^+^CD8^+^ DP T cells

**Figure 7:**
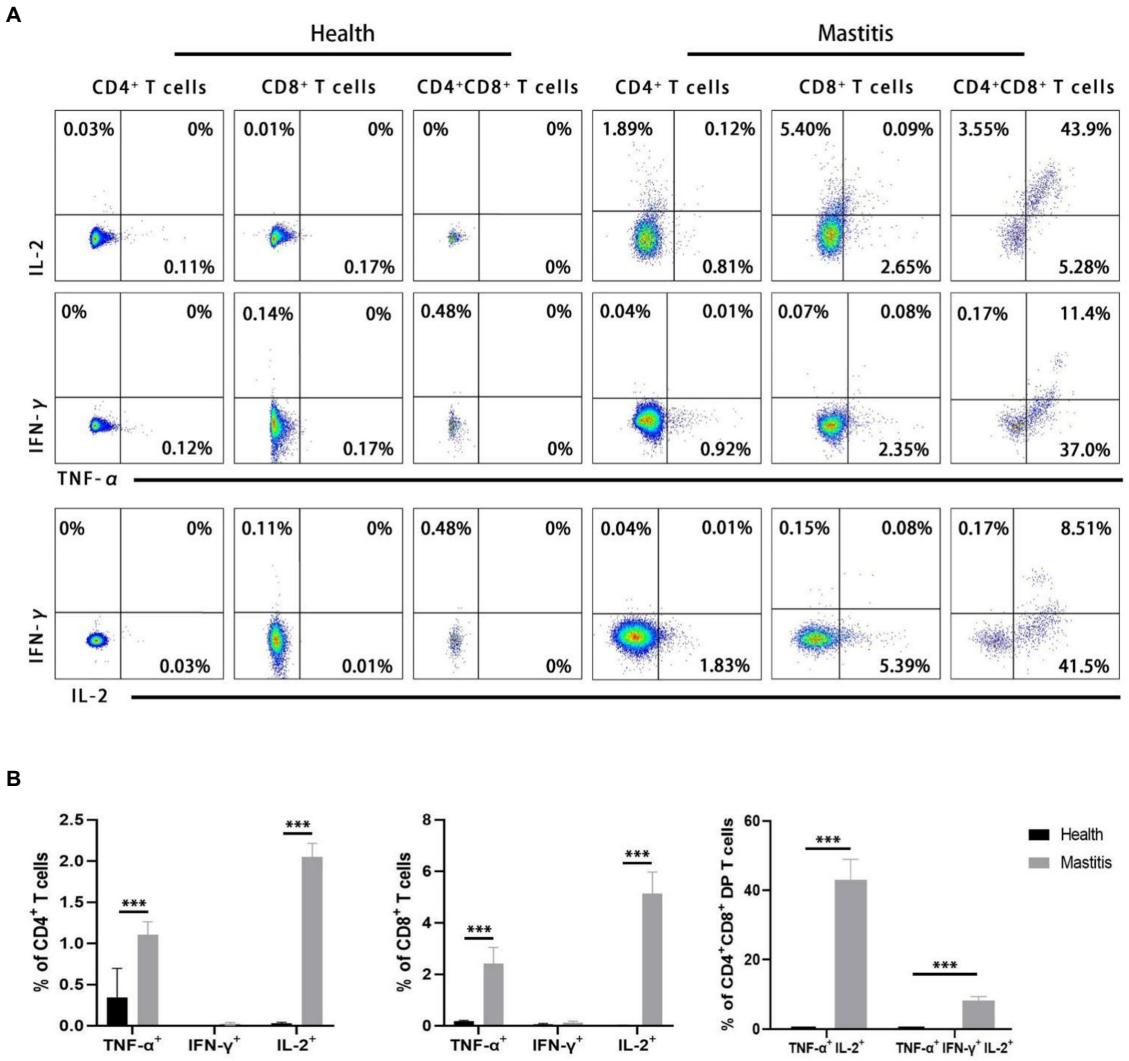
Identification of multifunctional TNF-α, IFN-γ and IL-2T cells in natural *B. contaminans* infection. **(A)** Representative plot of TNF-α, IFN-γ and IL-2 secreted by CD4^+^CD8^−^, CD8^+^CD4^−^ and CD8^+^CD4+ T cells. Boolean gating was applied in order to identify single-, double-, and triple-cytokine-producing T cells. **(B)** Percentage of CD4^+^CD8^−^, CD8^+^CD4^−^, and CD8^+^CD4^+^ T cells secreting single, dual, and triple cytokines.

Simultaneous expression of different cytokines at the level of individual T cells is considered to be a marker of protective immune response (Betts et al., 2006; Darrah et al., 2007; Kannanganat et al., 2007; Precopio et al., 2007; Akondy et al., 2009). Our interest therefore focused on the potential role of multifunctional CD4 and CD8 T cells expressing TNF-α, IFN-γ, and IL-2. In order to authenticate the potential multifunctional *B. contaminans*-specific T cells, intracellular cytokine staining was performed to label the expressions of TNF-α, IFN-γ, and IL-2 in CD4^+^, CD8^+^, and DP T cells following *B. contaminans in vitro* restimulation of PBMCs. As shown in Figure 7A, DP T cells infected with *B. contaminans* were identified as triple-cytokine producing cells in which IL-2 is produced in combination with TNF-α. However, there were no double-cytokine or triple-cytokine produced in CD4^+^ and CD8^+^ T cells. The CD4^+^, CD8^+^, and DP T cells of non-infected control animals hardly secrete the above cytokines in response to *B. contaminans* stimulation. The CD4^+^, CD8^+^ T cells of cows with mastitis expressed significantly higher single-cytokine (TNF-α or IL-2) than those of healthy cows. After natural infection with *B. contaminans*, the proportion of TNF-α/IL-2 double-producing and TNF-α/IFN-γ/IL-2 triple-producing DP T cells increased significantly (Figure 7B). Collectively, these above findings might support the hypothesis that multiple-cytokine-producing *B. contaminans*-specific CD4^+^CD8^+^ T cells are involved in clearing infection.

## Discussion

In the present study, an outbreak of clinical mastitis was investigated among a total of 650 lactating cows, microbiological analyses have demonstrated the causative agent of *B. contaminans*. Mastitis is a disorder of great economic significance, as it reduces both milk quality and quantity and increases the global demand for veterinary antibiotics (Filor et al., 2022). Additionally, the inherent resistance of *B. contaminans* isolates to the majority of antibiotics restricts treatment options for infected animals, resulting in additional losses from slaughtering infected animals. Attempts have been made to recognize the source of the infection during the epidemic described here. Since no new animals have been recently introduced to the herd, it is probable that the cows contracted the disease from an environmental source. Surprisingly, *B. contaminans* was unable to be cultivated from any of the environmental samples in the farm. In addition, of *B. contaminans* was identified from the cows’ milk with at least one prior lactation, but not from the cows’ milk experiencing their first lactation. Undoubtedly, the epidemics of *Pseudomonas aeruginosa* mastitis in cows is associated with the administration of a possibly contaminated dry-cow antibiotic infusion (Osborne et al., 1981). Therefore, it is probable that antibiotic-resistant bacteria produced by the dry-cow antibiotic medicationmay be a cause of infection. Regardless of the source, *Burkholderia* strains were probably spread to uninfected animals through the milking process after they were reintroduced into the herd (Berriatua et al., 2001).

In this study, *B. contaminans* was found to be resistant to many antibiotics tested (penicillin, streptomycin, lincomycin and amoxicillin). Previous studies have extensively investigated antibiotic resistance in *B. cepacia*. Resistance to β-lactam antibiotics such as penicillin and amoxicillin is caused by class A β-lactamases encoded by *B. cepacia* organisms, firstly described in the PenA-PenR system of *B. cepacia* 249, which are now named PenB and PenR (AmpR) (Rhodes and Schweizer, 2016). PenB is a Class A penicillinase with broad spectrum carbapenemase character, which is highly conserved in *B. cepacia* (Hwang and Kim, 2015). Resistance to aminoglycoside antibiotics such as streptomycin may be caused by AmrAB-OprM efflux pump expression of *B. contaminans* (Podnecky et al., 2015). In *B. vietnamiensis*, aminoglycoside resistance emerges during chronic infection or after *in vitro* exposure to aminoglycosides and is the result of AmrAB-OprM efflux pump expression, which is most likely a homolog of *B. cepacia* AmrAB-OprA (Jassem et al., 2014). To the best of our knowledge, this is the first report of lincomycin resistance in *Burkholderia*, and the underlying mechanisms need to be further studied. Similar to numerous other opportunistic infections, it seems that serious illness and mortality may ensue from infection with any *B. cepacia* species, depending on the clinical condition and propensity of persons with cystic fibrosis at the moment of infection. All *B. cepacia* species are indeed very resistant to antibiotics (Mahenthiralingam et al., 2008). Hence, treatment of persistent infections is extremely challenging.

During infection and after recurrent pathogen interactions, previously developed mechanisms of the adaptive immune system are activated (den Reijer et al., 2013; Karauzum and Datta, 2017). They include the participation of antibodies generated by B lymphocytes and cell-mediated immune responses governed by T lymphocytes. Typically, a bacterial infection is contained and eliminated after the development of pathogen-specific T and B cell immunity. Moreover, these immune responses are essential for protecting the host from subsequent infections (Kurtz et al., 2017). Remarkably, little is known about the precise function of the humoral immune response in *B. cepacia* infection. The most notable finding from a previous report (Grönlund et al., 2006) was the enhanced expression and proportion of B-lymphocytes in blood, non-infected and infected quarters in persistent sub-clinical mastitis caused by Gram-positive bacteria, such as *S. aureus*. The present study identified the reduced total number of B cells in the *B. contaminans* naturally-induced mastitis cows. Remarkably, we observed a negative correlation between the total number of CD21^+^ B cells and CFU. This indicates that the decrease in B-cell numbers is directly caused by *B. contaminans* infection. B lymphocytes can serve as antigen-presenting cells, and differentiate into plasma cells that produce immunoglobulins. Antibodies produced are beneficial in the opsonization and phagocytosis of bacteria (Nowicka and Grywalska, 2019). Taken together, this may explain the bad infective consequences of *B. cenocepacia*. In addition, prior studies have shown that the absolute number of B-lymphocytes in the blood dropped concurrently with the onset of mastitis produced by endotoxin and recovered with the remission of clinical symptoms (Ishikawa and Shimizu, 1983). LPS is a component found in the Gram-negative bacteria’ cell wall that is known as endotoxin. *Burkholderia cenocepacia*’s lipopolysaccharide (LPS) is likewise highly inflammatory (Govan, 2003) Therefore, we concluded that the decrease in B-cell number in mastitis cattle may be due to the high inflammatory level of LPS in *B. contaminans.*

Previous studies have shown that IgG, IgM and IgA isoforms of antibodies secreted by B cells are present in the peripheral blood of dairy cows, among which IgG and IgM contents were abundant, while IgA contents were scarce (Enger et al., 2021). As the staple antibody against bacterial cow mastitis, IgG is often used as a diagnostic marker due to its prolonged expression and high sensitivity (Bourry and Poutrel, 1996; Wellnitz et al., 2013; Wall et al., 2016; Drumm et al., 2022; Khatun et al., 2022). The IgM normally precedes IgG antibodies and appears temporarily in an acute infection and functioning in the primary antibody-mediated immune response (Wagner et al., 2008). In the inflammatory response caused by *Burkholderia*, IgG is a better indicator for early serodiagnosis than IgM with an overall higher sensitivity, specificity, positive/ negative predictive value and likelihood ratios (Hii et al., 2017). In this study, samples were collected at a certain time point, and were subsequently applied to detected the total IgG concentrations. The results showed that no significant difference was observed in total IgG concentration between the healthy cows and cows with mastitis. This suggests that at the sampling time point in this study, the levels *B.contaminans*-specific IgG may be too low to alter the total IgG concentrations. This result further confirmed our conjecture that humoral immune response may not be adequate to fight intracellular infection of *B.contaminans*. Therefore, to clarify the protective role of B cells during mastitis in cows infected with *B. contaminans*, we detected the expression of surface IgG in B cells. Further analysis of B cell subsets revealed that the frequency of IgG^+^ B cells was significantly higher in the *B.contaminans*-infected mastitis patients than in the healthy cows. IgG is produced by IgG^+^ B cells, which undergo differentiation into IgG-ASC upon activation. IgG^+^ B cell aberrations are associated with autoimmune disorders and persistent infections (Kong et al., 2016). We found that a considerably decreased proportion of CD27^+^IgG^+^ B cells (in total B cells) was observed in the *B. contaminans* naturally-induced mastitis cows, which was related to *B. contaminans* infection. Since CD27^+^IgG^+^ B cells belonged to the memory B cell compartments, we examined the prevalence of memory B cells across both groups, and the findings showed that the percentage of memory B cells in total B cells was increased in the *B. contaminans*-infected cows. This indicated that the elements responsible for the decrease of CD27^+^IgG^+^ B cells may be related to a reduction in memory B cells in *B. contaminans* infections.

Like B cells, the role of *B. cepacia*-specific T cells has not been well described. As *B. contaminan*s is an internal pathogen, it is considered that such responses are crucial, especially as antibodies specific to the species have minimal impact on the establishment of immunity (Raupach and Kaufmann, 2001). To better comprehend the host defense mechanism in mastitis, we have evaluated the alterations in T cell subset groups found in the blood of naturally *B. contaminans*-induced mastitis-stricken cows. We observed a significant increase in the total number of γδ T cells and CD4^+^ CD8^+^ DP T cells in the blood of infected animals compared to the blood of healthy animals. However, there was no difference in the percentage and number of CD4^+^ and CD8^+^ T cells in the blood between the two groups. The function of CD4^+^CD8^+^ DP T cells is mostly unknown. DP T cells were considered to constitute a developmental phase in the thymus prior to maturation into CD8^+^ or CD4^+^ (single positive) mature T cells (Germain, 2002). Consequently, in peripheral tissues and blood, the majority of T cells have maintained expression of just one of such co-receptors with distinct roles, with CD8 T cells directly engaged in cytotoxicity against tumor or infected cells and CD4 T cells orchestrating the immune response. Nonetheless, mature CD4^+^CD8^+^ DP T cells have been identified in peripheral tissues and blood in a variety of conditions, including human malignancies (Overgaard et al., 2015). These cells are diverse and/or exhibit pleiotropic activities, which must be explored in the context of each specific illness. Recent study indicates that DP T cells promote the functional polarization of naive CD4^+^ T cells toward a Th2 phenotype. This ability of DP T cells was detected in healthy donors and was amplified in patients with urologic malignancy, who had higher amounts of circulatory DP T cells (Bohner et al., 2019).

γδ T cells are considered as innate-like T cells with a limited pre-activated phenotype and TCR repertoire (Shiromizu and Jancic, 2018), and they are the major T-cell population in newborn ruminants’ circulation (Jörundsson et al., 1999). They may react promptly to infection or cytokine stimulation in a non-MHC-restricted manner, secreting a broad spectrum of cytokines and exhibiting direct cytotoxicity against infected and altered cells (Shiromizu and Jancic, 2018). It has been suggested that γδ T cells supplementαβ T cells in host defense by giving a fast reaction before theαβ T-cell response has completely formed, i.e., the first line of defense (Williams, 1998). Certain activated γδ T cells are also capable of presenting antigen to CD4^+^ T cells and priming bacterial antigen-specific CD8^+^ T cells. These cells also seem to generate costimulatory molecules in addition to cytokines, suggesting that γδ T cells may really influence the activity ofαβ T cells (Soltys and Quinn, 1999). Thus, the elevated amounts of γδ T cells in the blood of mastitis-affected cows are consistent with their hypothesized involvement in controlling the inflammatory response.

And on this basis we determined the percentages of CD27^+^ T cells and CD44^+^ T cells in the αβ or γδ T cells subsets of the healthy and *B. contaminans* naturally-induced mastitis cows to assessment the differentiation and activation of T cells. However, we did not find the differences in the percentages of CD27^+^ T cells in either WC1^+^ γδ, CD8^+^, and CD4^+^ T cells between the two groups. CD27, a co-stimulatory molecule, is often used to detect T cell development phases. Potentially, CD27 expression may be utilized to track γδ T cell response to *M. bovis* infections in cattle (Guerra-Maupome et al., 2019). Our results indicated that T cells from the *B. contaminans* infected cows shared a similar differentiation phenotype with those from the healthy cows at the early time-points. In addition, we determined the expression of CD44 molecule on the T subsets of the mastitis or healthy cows to investigate the activation of T subsets. We found that the percentages of activated WC1^+^ γδ T cells in peripheral blood were increased in the *B. contaminans* infected cows. However, there was no difference in the percentage of CD4^+^ CD44^+^ and CD8^+^CD44^+^ T cells in the blood between the two groups. The activation of T cells is one of the initial stages in the host’s response to infections, and the emigration of T cells from the circulation to areas of inflammation requires the progressive interaction of different adhesion molecules released by T cells (Odoardi et al., 2012). CD44, a leukocyte adhesion molecule, interacts with the extracellular matrix’s components and is regarded as necessary for T-cell extravasation in inflammatory locations. This adhesion molecule has been identified as a sensitive indication of T-cell activation (Bosworth et al., 1991). Thus, we conclude that γδ T cells are selectively activated during the host’s defense to *B. contaminans* in comparison to αβ T cells. And variations in the expression of such adhesion molecules on blood T cells may serve as a possible diagnostic sign of mastitis that can be examined quickly and inexpensively. Our research further supports the notion that γδ T cells supplementαβ T cells in host defense by giving a fast reaction prior to the αβ T-cell response has completely formed, i.e., the first line of defense.

IL-2, TNF-α, and IFN-γ have a vital role in mastitis prevention, according to bovine studies (Alluwaimi, 2004). Recent research on immune responses to infectious disorders has uncovered and characterized a vital function for multifunctional T cells that co-express IL-2, TNF-α, and IFN-γ. It has been hypothesized that multifunctional T cells are a characteristic of protective immunity. For instance, calves typically infected with bovine tuberculosis had a predominance of TNF-α^+^IFN-γ^+^IL-2^+^ and TNF-α^+^IFN-γ^+^IL-2^−^ CD4^+^ T cells with a TEM phenotype (Whelan et al., 2011). It should be noted that, due to the limitation of immunological reagents (the fluorescence of WC1 and TNF-α antibody is in conflict), the expression of activated γδ T inflammatory cytokines was not detected in our study. However, previous studies have shown that γδ T cells in peripheral blood of cows express little IFN-γ and do not express IL-2 in response to superantigen activation (Fikri et al., 2001). Further studies are required to determine the expression inflammatory factors in *B. contaminans*-specific γδ T cell. But executinging triple-cytokine staining of *in vitro* stimulation of PBMCs, we observed that many *B. contaminans*-specific CD4^+^CD8^+^ DP T cells can be activated to produce three of the cytokines TNF-α, IFN-γ and IL-2 simultaneously. This early appearance in regard to the clearance of clinical signs confirms their immediate role in the primary immune response against *B. contaminans*. After infection with *B. contaminans*, CD4^+^ or CD8^+^ T cells isolated from blood of cows produce hardly any IFN-γ. This might indicate that IFN-γ is not directly involved in the host immune response in the early stage of mastitis caused by *B. contaminans* in cows. In this study, individual TNF-α- producing or IL-2-producing CD4 and CD8 cells were a relatively small population. The cows were infected for *circa* 14 days. It may be not enough time to develop the cell immune response.

In conclusion, we have investigated the lymphocyte subsets distributions and proinflammatory cytokine’s expression on leukocytes isolated from the blood of cows with spontaneously acquired mastitis caused by *B. contaminans* and healthy cows. Our studies showed a significant reduction in the percentages and absolute number of CD21^+^ B cells in cows with *B. contaminans* naturally-induced mastitis. And we have demonstrated that γδ T cells and CD4^+^CD8^+^ DP T cells significantly increased at the early stage after infection and displayed differential activation and differentiation. In addition, our results indicated the production of *B. contaminans*-specific TNF-α^+^IFN-γ^+^IL-2^+^ DP T cells in the immunological response of cows to Gram-negative bacteria infection. This comprehensive analysis of bovine T, B cells responses to infection with *B. contaminans* will aid in the development of more effective therapies or vaccinations against bovine mastitis.

## Data availability statement

The data presented in the study are deposited in the GenBank repository, accession number OP890178.

## Ethics statement

The animal study was reviewed and approved by Institutional Animal Care and Use Committee of Yangzhou University.

## Author contributions

ZZ, ZY, and YYang contributed to conception and design of the study. ZZ, YYao, HJ, YM, WC, FZ, and KW performed the experiments. ZZ, JY, CY, JS, and YYang organized the database. ZZ, CY, and JS performed the statistical analysis. ZZ wrote the first draft of the manuscript. YYang revised the manuscript. All authors contributed to the article and approved the submitted version.

## Funding

This study was financially supported by Seed Industry Vitalization Program of Jiangsu Province (JBGS[2021]117), National Natural Science Foundation of China (32002263 and 31872324), Jiangsu Agriculture Science and Technology Innovation Fund (JATS[2021]486), Basic Research Program of Jiangsu Province (BK20190881), Postdoctoral Research Foundation of China (2019M650126), Young Elite Scientists Sponsorship Program of Jiangsu Province (TJ-2022-031), the 111 Project D18007 and Project Funded by the Priority Academic Program Development of Jiangsu Higher Education Institutions (PAPD).

## Conflict of interest

The authors declare that the research was conducted in the absence of any commercial or financial relationships that could be construed as a potential conflict of interest.

## Publisher’s note

All claims expressed in this article are solely those of the authors and do not necessarily represent those of their affiliated organizations, or those of the publisher, the editors and the reviewers. Any product that may be evaluated in this article, or claim that may be made by its manufacturer, is not guaranteed or endorsed by the publisher.
